# Resveratrol contributes to NK cell-mediated breast cancer cytotoxicity by upregulating ULBP2 through miR-17-5p downmodulation and activation of MINK1/JNK/c-Jun signaling

**DOI:** 10.3389/fimmu.2025.1515605

**Published:** 2025-02-03

**Authors:** Bisha Ding, Jie Li, Jia-Lin Yan, Chun-Yan Jiang, Ling-Bo Qian, Jie Pan

**Affiliations:** ^1^ Cancer Center, Department of Medical Oncology, Zhejiang Provincial People’s Hospital, Affiliated People’s Hospital, Hangzhou Medical College, Hangzhou, Zhejiang, China; ^2^ School of Basic Medical Sciences and Forensic Medicine, Hangzhou Medical College, Hangzhou, Zhejiang, China

**Keywords:** NK cell, ULBP2, cytotoxicity, MINK1, JNK, resveratrol, breast cancer

## Abstract

**Backgrounds:**

Natural killer (NK) cell mediated cytotoxicity is a crucial form of anti-cancer immune response. Natural killer group 2 member D (NKG2D) is a prominent activating receptor of NK cell. UL16-binding protein 2 (ULBP2), always expressed or elevated on cancer cells, functions as a key NKG2D ligand. ULBP2-NKG2D ligation initiates NK cell activation and subsequent targeted elimination of cancer cells. Enhanced expression of ULBP2 on cancer cells leads to more efficient elimination of these cells by NK cells. Resveratrol (RES) is known for its multiple health benefits, while current understanding of its role in regulating cancer immunogenicity remains limited. This study aims to investigate how RES affects the expression of ULBP2 and the sensitivity of breast cancer (BC) cells to NK cell cytotoxicity, along with the underlying mechanisms.

**Methods:**

The effects of RES on ULBP2 expression were detected with qRT-PCR, western blot, flow cytometry analysis and immunohistochemistry. The effects of RES on sensitivity of BC cells to NK cell cytotoxicity were evaluated *in vitro* and *in vivo*. The target gene of miR-17-5p were predicted with different algorithms from five databases and further confirmed with dual-luciferase reporter assay. Overexpression and knockdown experiments of miR-17-5p and MINK1 were conducted to investigate their roles in regulating ULBP2 expression and subsequent JNK/c-Jun activation. The JNK inhibitor sp600125 was utilized to elucidate the specific role of JNK in modulating ULBP2 expression.

**Results:**

RES increased ULBP2 expression on BC cells, thereby augmenting their vulnerability to NK cell-mediated cytotoxicity both *in vitro* and *in vivo*. RES administration led to a reduction in cellular miR-17-5p level. MiR-17-5p negatively regulated ULBP2 expression. Specifically, miR-17-5p directly targeted MINK1, leading to its suppression. MINK1 played a role in facilitating the activation of JNK and its downstream effector, c-Jun. Furthermore, treatment with sp600125, a JNK inhibitor, resulted in the suppression of ULBP2 expression.

**Conclusions：:**

RES potentiates ULBP2-mediated immune eradication of BC cells by NK cells through the downregulation of miR-17-5p and concurrent activation of the MINK1/JNK/c-Jun cascade. This finding identifies RES as a potentially effective therapeutic agent for inhibiting BC progression and optimizing NK cell-based cancer immunotherapy.

## 1 Introduction

Resveratrol (RES), a naturally occurring polyphenol compound, is present in over 70 widely distributed dietary and medical plants species such as peanuts, grapes, berries and white hellebore (1). In recent decades, RES has garnered significant attention for its wide array of well-documented health benefits, including antioxidant, anti-aging, anti-inflammatory, and anticancer properties (2). The potential of RES as an anticancer agent is well-established through an extensive array of preclinical studies (3–5). Breast cancer, one of the most prevalent cancers among women globally, can potentially be prevented through RES by mechanisms that include inhibiting cell proliferation, inducing cell cycle arrest and apoptosis, reducing inflammatory responses, suppressing angiogenesis, and facilitating epigenetic modifications (6–8). Of particular interest, RES has demonstrated its ability to strengthen the immune system, thereby enhancing antitumor immunity. This is achieved through several mechanisms such as attenuating resistance in cancer stem cells, inhibiting the production of immunosuppressive cytokines, and alleviating immunosuppression within the tumor microenvironment (9, 10). However, the mechanism underlying cancer immunomodulation by RES remains fragmentary and deserves more studies.

Natural killer group 2 member D ligands (NKG2DLs) are important targets in cancer immunomodulation. They consist of eight surface glycoproteins, namely MICA and MICB (major histocompatibility complex class I chain-related proteins A and B), and ULBP1-6 (UL16-binding proteins 1-6) (11). NKG2DLs are the specific ligands of natural killer group 2 member D (NKG2D) receptor, a key activating receptor prominently featured on natural killer (NK) cells (12). NKG2DLs, typically either undetectable or low expressed on healthy cells, can be induced in the progression of cellular malignant transformation (11). The ligation of NKG2DL and NKG2D leads to NK cell activation, subsequently enabling the killing of cancer cells. This process is a pivotal mechanism of immunosurveillance and immune clearance. In breast cancer, the upregulation of NKG2DLs was shown to be associate with cancer suppression (13–15).

NKG2D initiates cytotoxicity against cancer cells that display NKG2DLs on their surface. However, malignantly transformed cells often reduce or downregulate the expression of surface NKG2DLs to evade the immune clearance. Cancer cells manipulate the expression of these ligands at both transcriptional and post-transcriptional levels. Several transcription factors, including krüppel-like factor 4 (KLF4), nuclear factor kappa-B (NF-κB) and c-Myc, are documented to function in NKG2DL transcription (16–18). The growth and metastasis of breast cancer in mice were enhanced by the downregulation of NKG2DL expression, with glycogen synthase kinase-3β (GSK-3β) contributing to these processes (14). Our earlier investigation has demonstrated that the downregulation of MICA/B and ULBP2 by some NKG2D ligand-targeting microRNAs (miRNAs) resulted in a diminished NK cell-mediated cytotoxic response against breast cancer cells (15). And shedding or internalization is also involved in the reduction of the surface ligands (19). Given the significant role that NKG2DLs play in tumoricidal activity, they have attracted considerable interest as promising candidates for designing innovative cancer preventive and immunotherapeutic strategies. Pharmacological interventions, such as metformin, decitabine, and valproic acid, have been shown to effectively increase NKG2DL levels, thereby enhancing the immune clearance of cancer cells (15, 20, 21).

Herein, RES was found to effectively upregulate the expression of ULBP2, a pivotal component of NKG2DLs, thereby augmenting the cytolytic activity of NK cells against breast cancer cells both *in vitro* and *in vivo*. Mechanistically, RES suppressed the oncogenic miR-17-5p, which directly targets misshapen like kinase 1 (MINK1). MINK1 activates ULBP2 through its downstream JNK/c-Jun signaling cascade. A high surface expression level of ULBP2 significantly facilitates the recognition and subsequent elimination of breast cancer cells by NK cells.

## 2 Materials and methods

### 2.1 Cell culture and reagents

The cell lines (MDA-MB-231, BCap37, MCF7, MDA-MB-468 and HeLa) were obtained from the Cell Bank of the Chinese Academy of Sciences (Shanghai, China). MDA-MB-231 and MDA-MB-468 are triple-negative breast cancer cell lines, whereas BCap37 and MCF7 are estrogen receptor-positive breast cancer cell lines. MDA-MB-231, MDA-MB-468 and HeLa cells were maintained in Dulbecco’s modified Eagle’s medium (DMEM, Gibco, Life technologies, Carlsbad, CA, USA). BCap37and MCF-7 cells were cultured in Roswell Park Memorial Institute 1640 medium (Gibco, USA). The media were supplemented with 10% fetal bovine serum (Gibco, USA) and 1% streptomycin/penicillin antibiotics, and the cultures were incubated at 37°C under a 5% CO_2_ atmosphere. RES (Selleck Chemicals, Houston, TX, USA) was solubilized in dimethyl sulfoxide (DMSO) to yield a 109.5 mmol/L stock solution, which was then stored at -20°C. The cells were exposed to various concentrations of RES (6.25, 12.5, or 25 μmol/L) for a duration of 48 hours.

### 2.2 Plasmids and cell transfection

The overexpression plasmid of MINK1(pcDNA3.1-MINK1), pcDNA3.1 control and siRNAs of MINK1 (si-MINK1-1, si-MINK1-2), SQSTM1 (si-SQSTM1-1, si-SQSTM1-2), CDKN1A (si-CDKN1A-1, si-CDKN1A-2) were obtained from Repbio (Hangzhou, China). The mimic, inhibitor and negative controls (NC) of miR-17-5p were bought from Ribobio (Guangzhou, China). The siRNA sequences were listed in **Table 1**. The aforementioned siRNA, plasmids and miRNA mimics/inhibitors were introduced into their respective target cells using Lipofectamine 3000, following the previously described transfection protocol (22).

**Table 1:** Sequence of siRNA.

siRNA	Sequence
si-MINK1-1	GGAACAAGAUUCUGCACAA
si-MINK1-2	GAAAGAGGAGACAGAAUAU
si-SQSTM1-1	CUUCCGAAUCUACAUUAAA
si-SQSTM1-2	GAAUCUACAUUAAAGAGAA
si-CDKN1A-1	AGUUUGUGUGUCUUAAUUA
si-CDKN1A-2	GCUUAGUGUACUUGGAGUA

### 2.3 RNA extraction and quantitative reverse transcription-polymerase chain reaction

Total RNA was isolated from cells using RNAiso Plus (TaKaRa, Kusatsu, Japan) and then subjected to reverse transcription into complementary DNA (cDNA) utilizing the PrimeScriptTM RT Reagent Kit (#RR037A, TaKaRa). For conventional RNA RT-PCR, random hexamer primers were employed, whereas specific primers (Ribobio) were utilized for miRNA RT-PCR. The expressions of ULBP2, MINK1, CDKN1A and SQSTM1 were quantified using qRT-PCR with GAPDH as internal control. The sequences of specific primers were provided in **Table 2**. Expression level of miR-17-5p was quantified using a BulgeLoop miRNA qRT-PCR primer set (Ribobio), with U6 serving as an endogenous reference for normalization. The qRT-PCR assays were carried out on a LightCycler 480II system (Roche Diagnostics, Basel, Switzerland), employing the SYBR Premix EX Tag Kit (#RR420A, TaKaRa). Relative expression levels of both miRNA and RNA were determined using the 2^-ΔCt^ method, following normalization to a reference control.

**Table 2:** Primers for quantitative RT-PCR.

Primer	Sequence
MINK1	FP: GGAGGACTGTATCGCCTATATCT RP: GTCTCGATGGATCACCTTGTG
CDKN1A	FP: TGTCCGTCAGAACCCATGC RP: AAAGTCGAAGTTCCATCGCTC
SQSTM1	FP: GCACCCCAATGTGATCTGC RP: CGCTACACAAGTCGTAGTCTGG
ULBP2	FP: CAGAGCAACTGCGTGACATT RP: GGCCACAACCTTGTCATTCT
ULBP5	FP: ACAGGATGGCTTGAGGACTTCTTG RP: TGATGAGGAGGCAGCAAAGGATG
ULBP6	FP: GCTTCATCCTCCCTGGCATCTG RP: GGCTGCTGGACATACACCGTAG
GAPDH	FP: CTGGGCTACACTGAGCACC RP: AAGTGGTCGTTGAGGGCAATG
U6	FP: CTCGCTTCGGCAGCACATA RP: AACGCTTCACGAATTTGCG

### 2.4 Western blot

The cells subjected to pretreatment were lysed in radioimmunoprecipitation assay buffer for total protein extraction. The protein concentrations were subsequently measured with a bicinchoninic acid (BCA) protein assay kit (Beyotime Biotec, China). Equivalent quantities of denatured protein samples (40 μg) were subjected to separation via 4%–20% SDS-polyacrylamide gel electrophoresis and then transferred onto polyvinylidene fluoride membranes. Following a 1-hour blocking step at room temperature with 5% skim milk (Yili, Hohhot, China) in tris-buffered saline containing 0.1% Tween-20, the membranes were incubated with the specific primary antibodies detailed in **Table 3** at 4°C overnight. GAPDH served as an internal control for normalization purposes. The membranes were subsequently incubated with an optimally diluted solution of the corresponding secondary antibodies conjugated to horseradish peroxidase for a duration of 2 hours at room temperature. Finally, the immunoreactive bands were visualized utilizing an enhanced chemiluminescence detection system (Thermo Scientific, Darmstadt, Germany).

**Table 3:** Antibodies for western blot and flow cytometry analysis.

Antibodies	Application	Source	Identifier
GAPDH	WB	abcam	ab181602
ULBP-2	WB	abcam	ab275023
MINK1	WB	Proteintech	13137-1-AP
JNK	WB	abcam	ab179461
Phospho-JNK	WB	CST	4668
c-Jun	WB	abcam	ab40766
Phospho-c-Jun	WB	abcam	ab32385
Anti-human ULBP2/5/6 PE	Flow Cyt	R&D systems	FAB1298P
Mouse IgG2A PE-conjugated Antibody	Flow Cyt	R&D systems	IC003P

### 2.5 Flow cytometry analysis

After pretreatment, the cells were rinsed with phosphate-buffered saline (PBS) and then incubated with the antibodies specified in **Table 3** for 25 minutes at 4°C in the dark. The flow cytometry assays were conducted using a BD FACSCalibur flow cytometer (BD Biosciences, San Jose, California, USA), with data acquisition and interpretation carried out utilizing the CellQuest software. The cells were gated based on their higher forward scatter and lower side scatter; characteristics typically indicative of live cells. No additional specific protocols were implemented to explicitly exclude dead cells. The mean fluorescence intensity (MFI) change (denoted as ΔMFI) was determined using the following formula: ΔMFI= (MFIwith specific mAb​−MFIwith isotype control​) ÷MFIwith isotype control​. To further facilitate comparison between the ΔMFI observed for a specific experimental treatment and that of a control treatment, the relative MFI (rMFI) was computed as follows: rMFI=ΔMFIspecific treatment/​ΔMFIcontrol treatment​​×100% (23).

### 2.6 Dual-luciferase reporter assay

The wild-type (WT) and mutant (MUT) 3′-UTR sequences of *ULBP2* were individually cloned into the pmirGLO vector (Repbio). HEK-293 T cells were cultured into 96-well plates. After 24 hours, the cells were co-transfected with 100 ng of either wild-type or mutant reporter plasmids, along with 100 nmol of miR-17-5p mimics or NC, for a duration of 6 hours. Forty-eight hours later, the Firefly/Renilla luciferase activities were quantified using the Dual Luciferase Reporter Assay Kit as described in the manufacturer’s instructions (Promega, Madison, WI, USA).

### 2.7 NK cell cytotoxicity assay *in vitro*

MDA-MB-231 cells were subjected to treatment with either 12.5 μM or 25 μM RES for a duration of 48 hours and subsequently seeded into a round-bottom 96-well plate. Before the cytotoxicity assay, the NK-92MI cell line was exposed to either PBS or anti-NKG2D antibody (50 mg/mL, Novus Biologicals, Littleton, CO, USA) for a period of 1 hour. Next, the effector NK-92MI cells were added to the respective wells at varying effector-to-target cell ratios of 10:1, 5:1, and 2.5:1. Following co-incubation at 37°C under an atmosphere containing 5% CO_2_ for a period of 4 hours, the supernatant was collected and subjected to analysis utilizing the CytoTox 96 NonRadioactive Cytotoxicity Kit (Promega). According to the protocols provided in the instruction manual for the Kit, damaged cells release intracellular lactate dehydrogenase (LDH), so the level of LDH is proportional to the number of the damaged cells. The LDH release is quantified by measuring the absorbance at 490 nm. The cytotoxic potential of the effector cells towards the target cells was quantified using the following formula: Cytotoxicity (%) = (LDH Release _Experimental_ - LDH Release _Effector spontaneous_ - LDH Release _Target spontaneous_)/(LDH Release _Target maximum_ - LDH Release _Target spontaneous_) ×100. The spontaneous LDH release from effector cells and target cells was assessed to mitigate any potential influence of LDH spontaneously released by NK cells and breast cancer cells on the experimental outcomes. The difference “LDH Release _Target maximum_ - LDH Release _Target spontaneous_” corresponds to the LDH release resulting from 100% lysis of all breast cancer cells, indicating the amount of LDH released when all breast cancer cells are completely damaged or killed.

### 2.8 Acute lung clearance assay

C57BL/6 male mice, aged 8 to 9 weeks, were allocated into three distinct groups and intraperitoneally administered one of the following treatments: anti-NK1.1 antibodies (dose: 300 μg per mouse, Clone PK136, 108759, Biolegend, San Diego, CA, USA), anti-mouse NKG2D monoclonal antibodies (dose: 300 μg per mouse, Clone 191004, MAB1547, Novus Biologicals), or IgG isotype control (dose: 300 μg per mouse, Clone 20116, MAB004, Novus Biologicals). Twenty-four hours later, the pretreated MDA-MB-231 cells were labeled with CFSE (Invitrogen). HeLa cells, which served as an internal control due to their relative insensitivity to killing by mouse NK cells, were labeled with PKH26 (Invitrogen). A suspension of the labeled cells (5×10^6^ cells of each population) was prepared by mixing them in 1 mL of PBS. Subsequently, 0.4 mL of this cell mixture was intravenously injected into the tail vein of each mouse. Five hours later, the lungs were excised and processed to generate single-cell suspensions suitable for flow cytometric analysis. The ratio of the tested target MDA-MB-231 cells to the control HeLa cells within these lung suspensions was then calculated. All animal experiments were conducted in strict compliance with the ethical guidelines set forth by the Ethics Committee for the Use of Experimental Animals in Hangzhou Medical College and adhered to the principles outlined in the Guide for the Care and Use of Laboratory Animals published by the US NIH (the 8th Edition, NRC 2011).

### 2.9 Immunohistochemistry in a xenograft model

MDA-MB-231 cells were subcutaneously inoculated into the right hind flanks of female BALB/c (nu/nu) immunodeficient mice. Once the tumors attained an approximate volume of 40 mm³, the mice were randomly allocated into three distinct groups. RES was dissolved in a vehicle solution composed of DMSO, polyethylene glycol 400, and distilled deionized water at a ratio of 1:1:3. The RES solution was administered intraperitoneally at daily dosages of either 25 mg/kg or 100 mg/kg for a continuous four-week period. The control group received an equivalent volume of the vehicle alone. At last, the mice were euthanized. The tumors were promptly fixed in 4% paraformaldehyde obtained from Sinopharm Chemical Reagent Co. (Shanghai, China), followed by tissue processing and sectioning. Paraffin-embedded sections of the xenograft tumor tissues, with a thickness of 4 μm, were prepared and utilized for immunohistochemical (IHC) staining. The slides were subjected to IHC using an anti-ULBP2 antibody (MA5-29636, Invitrogen, Carlsbad, CA, USA). Staining was visualized under an Olympus optical microscope.

### 2.10 Bioinformatic analysis

The target genes of miR-17-5p were computationally predicted using distinct algorithms from five databases: miRanda(http://www.microrna.org/microrna/home.do), TargetScan(http://www.targetscan.org), miRmap(https://mirmap.ezlab.org), PITA (http://genie.weizmann.ac.il/pubs/mir07/mir07_data.html) and picTar(https://pictar.mdc-berlin.de). The results of Kyoto Encyclopedia of Genes and Genomes (KEGG) pathway enrichment analysis for the shared predicted target genes were visualized utilizing the R package clusterProfiler from the Bioconductor project. Statistical significance was set at an adjusted p-value threshold of <0.05. mRNA and miRNA expression data for breast cancer samples (N=1104; Subtype: Luminal A 43.03%, Luminal B 19.11%, Triple negative 16.49%, Her2(+) 8.24%, NA 13.13%; Sex: Female 98.82%, Male 1.18%) were obtained from The Cancer Genome Atlas (TCGA) database (https://cancergenome.nih.gov/). Patient characteristics of breast cancer samples were shown in **Supplementary Figure 1**. According to the information provided by TCGA, mRNA and miRNA expressions were measured using IIIumina HiSeq 2000 Sequencing. Differential expression and correlation analyses were performed using the R package edgeR.

### 2.11 Statistical analysis

Statistical tests were performed and analyzed using Microsoft Excel and GraphPad Prism 9.0 software (San Diego, CA, USA). All data from a minimum of three independent experiments were expressed as mean ± standard deviation (SD). In the statistical analysis comparing two groups, the Shapiro-Wilk test was first used to assess the normality of the data. For non-normally distributed data, the non-parametric Mann-Whitney U test was employed to compare the two groups. For normally distributed data, an F test was conducted to compare variances between the groups. When both normality and equal variances were confirmed, an unpaired two-tailed Student’s t-test was used to determine statistical significance. If the variances were found to be unequal, the Welch’s t-test (t-test with Welch’s correction) was applied instead. A *p*-value threshold of <0.05 was adopted to denote statistical significance, represented graphically as* for *p*<0.05, ** for *p*<0.01, and *** for *p*<0.001.

## 3 Results

### 3.1 Resveratrol upregulates the expression of ULBP2 in breast cancer

**Figure 1:**
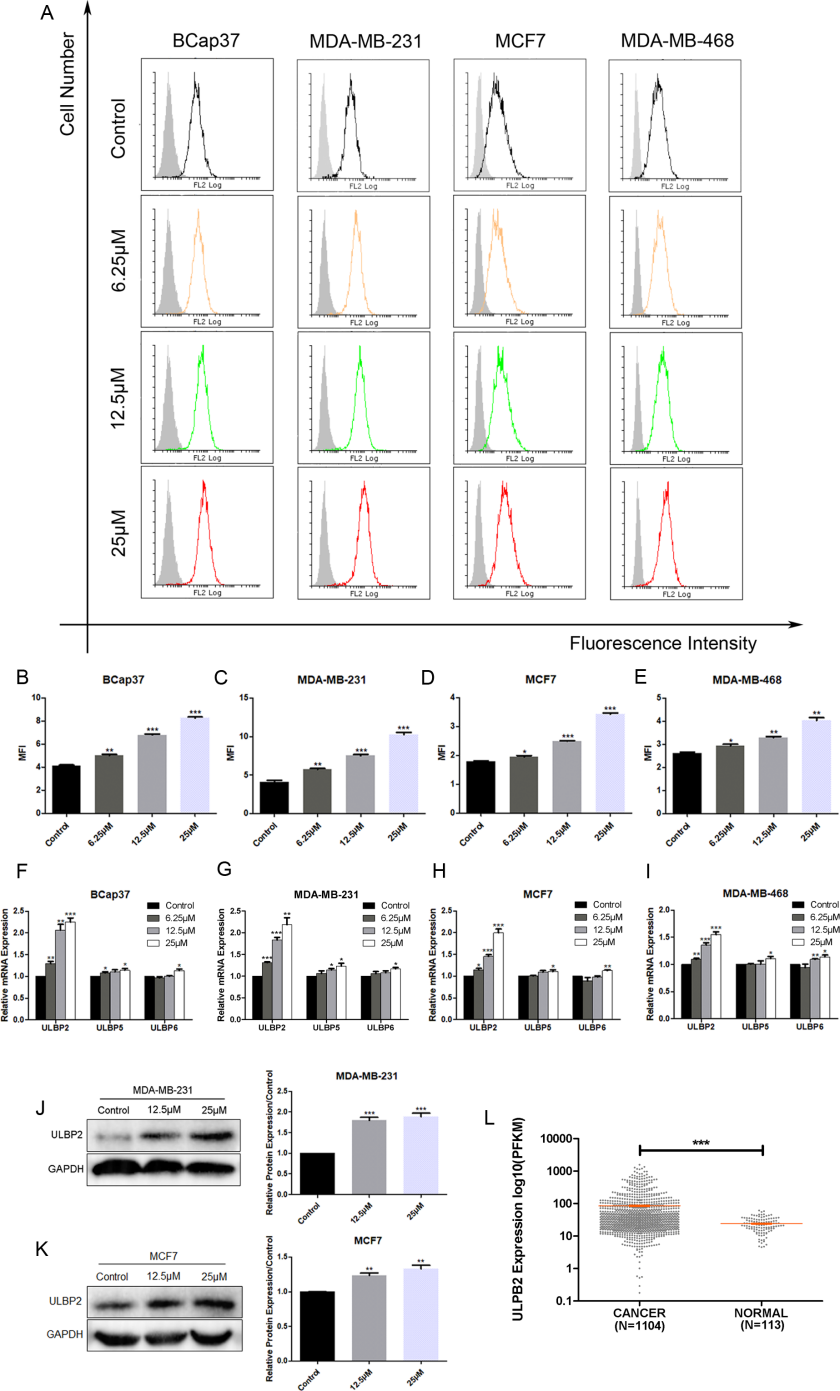
Resveratrol (RES) upregulates the expression of ULBP2 in breast cancer cells. Breast cancer cell lines (BCap37, MDA-MB-231, MCF-7 and MDA-MB-468) were treated with various concentrations of RES or control for 48 hours. **(A-E)** The surface protein levels of ULBP2/5/6 on BCap37, MDA-MB-231, MCF-7 and MDA-MB-468 cells were detected by flow cytometry. **(A)** are the depicted representative results from **(B-E)**. **(F-I)** The mRNA expression levels of ULBP2, ULBP5 and ULBP6 were detected in RES pretreated BCap37, MDA-MB-231, MCF-7 and MDA-MB-468 cells by qRT-PCR, with GAPDH as a reference. **(J, K)** The expression of ULBP2 protein was determined in RES pretreated MDA-MB-231and MCF-7 cells by Western blot (left) and the blots were further quantified with ImageJ (right). GAPDH was used as a loading control. **(L)** The differential expression of ULBP2 between breast cancer samples (n=1104) and adjacent normal breast tissue samples (n=113) from The Cancer Genome Atlas dataset was analyzed using Welch’s t-test. The unpaired two-tailed Student’s t-test was used to determine statistical significance in **(B-K)**, *p<0.05, **p<0.01, ***p<0.001 versus Control.

To investigate the effect of RES on NKG2DL expression in breast cancer, four distinct cell lines—MDA-MB-231, Bcap37, MCF7, and MDA-MB-468—were exposed to RES at concentrations of 6.25 μM, 12.5 μM, or 25 μM, as well as to a vehicle control, for a period of 48 hours. Our flow cytometric analysis demonstrated a dose-dependent increase in the MFI due to an antibody specific for ULBP2, ULBP5, and ULBP6 in cells treated with RES. This suggests that the expression of one or more of these ligands was upregulated in response to RES treatment (**Figures 1A–E**). To precisely determine which ligands are modulated by RES, we quantified mRNA expression levels in RES-treated and control cells using qRT-PCR with primers specific to each ULBP. Among all the *ULBP* genes analyzed, it was revealed that ULBP2 exhibited a consistent, pronounced, and dose-dependent increase in mRNA expression following RES treatment (**Figures 1F–I**). Moreover, the results from western blot assays demonstrated that exposure to RES led to a significant elevation in ULBP2 protein levels in both the MDA-MB-231 and MCF7 cell lines (**Figures 1J, K**). This finding corroborates the observed increase in ULBP2 mRNA expression, indicating that RES increases ULBP2 expression at both the transcriptional and translational levels in these cell lines.

ULBP2 is always undetectable or low-expressed on healthy cells, while it can be induced at the onset of malignant transformation. To assess the differential expression of ULBP2 between normal and neoplastic breast tissues, we conducted an analysis of data obtained from TCGA. Our findings revealed that the mean value of ULBP2 expression was significantly elevated in breast cancer specimens (n=1104) compared to a cohort of adjacent normal breast tissue samples (n=113) (**Figure 1L**).

### 3.2 MiR-17-5p which is suppressed in RES treated breast cancer cells inhibits ULBP2 expression

**Figure 2:**
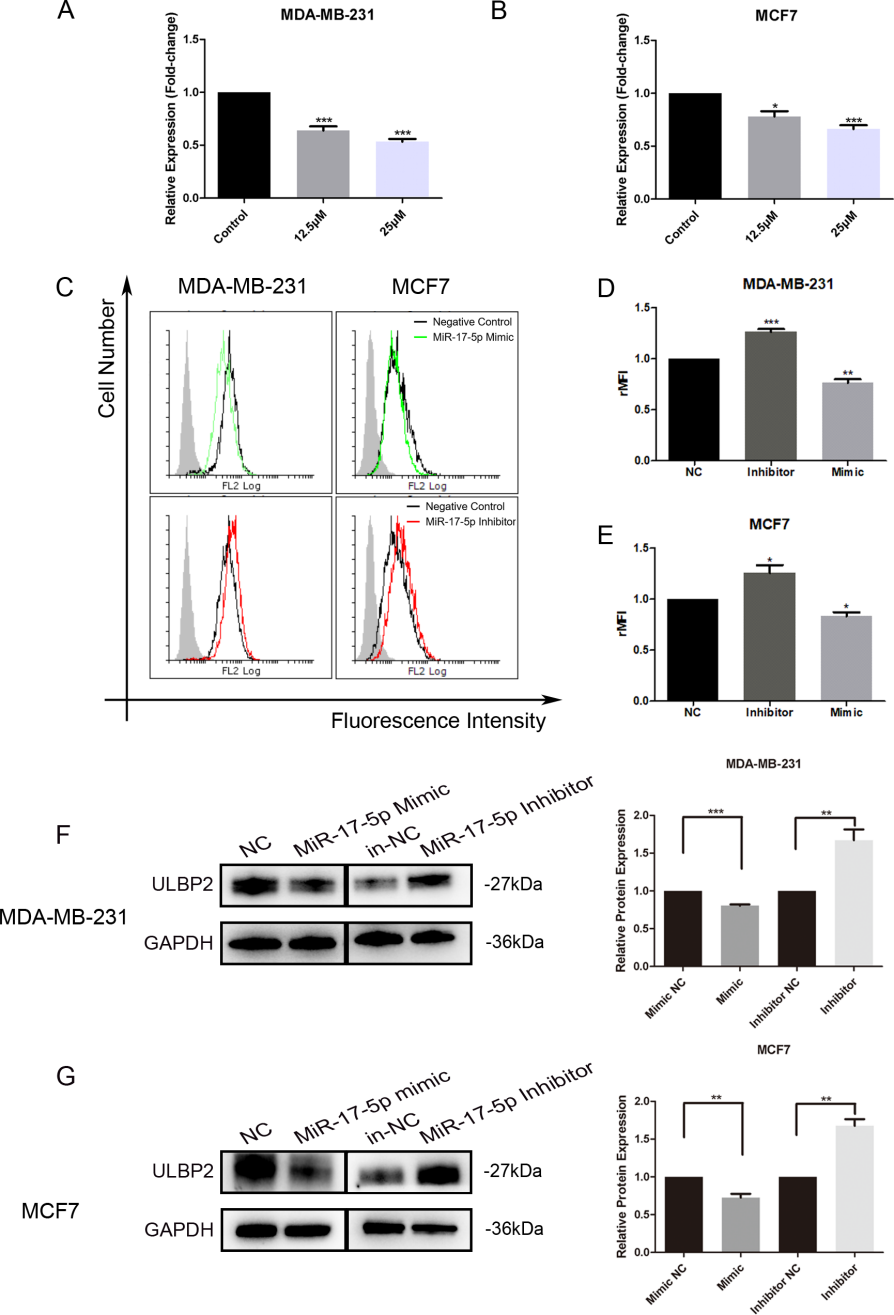
MiR-17-5p which is suppressed in RES treated breast cancer cells inhibits ULBP2 expression. **(A, B)** The breast cancer cells were treated with RES or control medium for 48 hours. The level of miR-17-5p was assessed with qRT-PCR, with U6 as a reference. **(C-G)** The breast cancer cells were transfected with miR-17-5p mimic, inhibitor or negative control (NC), respectively. **(C-E)** The expression of ULBP2/5/6 was determined by flow cytometry. **(C)** are depicted representative results from **(D, E)**. **(F, G)** The level of ULBP2 protein was detected by Western blot (left) and the blots were further quantified with ImageJ (right). GAPDH was used as a loading control. The unpaired two-tailed Student’s t-test was used to determine statistical significance. *p<0.05, **p<0.01, ***p<0.001 versus Control or NC.

To elucidate the molecular mechanism underlying ULBP2 regulation by RES, level of miR-17-5p was determined in RES treated breast cancer cells. When MDA-MB-231 and MCF7 cells were exposed to RES at concentrations of either 12.5 μM or 25 μM for 48 hours, a dose-dependent decrease in miR-17-5p levels was observed (**Figures 2A, B**), which substantiated the suppressive influence of RES on miR-17-5p expression.

To determine the regulatory role of miR-17-5p on ULBP2 expression, we transiently transfected MDA-MB-231 and MCF7 cells with either a miR-17-5p mimic, a miR-17-5p inhibitor, or a negative control (NC) oligonucleotide. With flow cytometry assays, the surface ULBP2/5/6 proteins were shown to be increased on miR-17-5p inhibitor-transfected MDA-MB-231 cells, while decreased on mimic-transfected cells. A similar pattern of observation was noted in MCF7 cells as well (**Figure 2C–E**). Western blot analysis revealed that the protein level of ULBP2 was consistently downregulated upon transfection with miR-17-5p mimics, while correspondingly upregulated following miR-17-5p inhibitor transfection in both MDA-MB-231 and MCF7 cell lines (**Figures 2F, G**). Thus, these observations indicate that RES suppresses the expression of miR-17-5p, and miR-17-5p negatively regulates the expression of ULBP2 in breast cancer.

### 3.3 *MINK1* is predicted as the target gene of miR-17-5p in ULBP2 regulation

**Figure 3:**
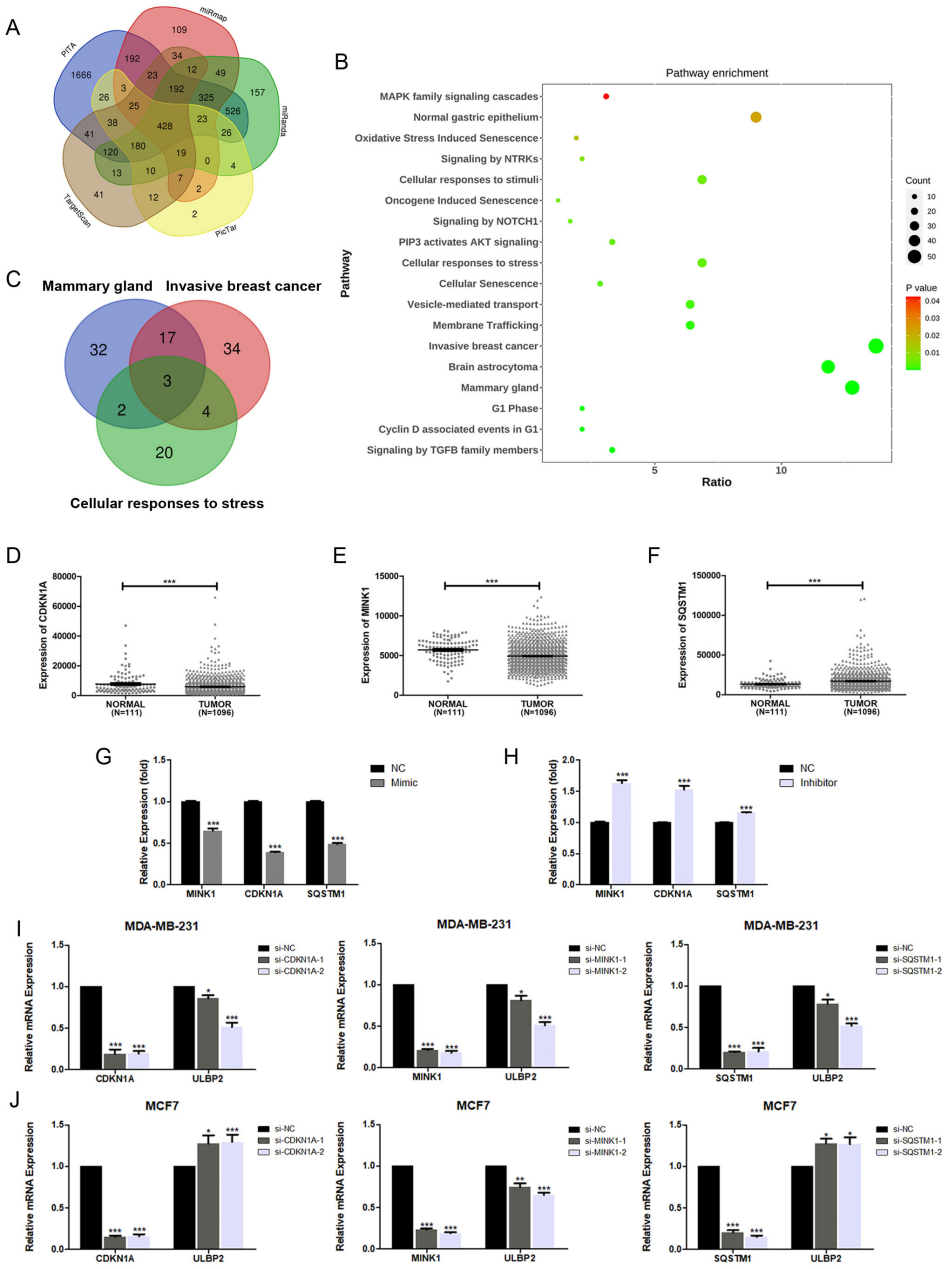
The target gene screening of miR-17-5p in ULBP2 regulation. **(A)** 428 genes were predicted as direct targets for miR-17-5p using PITA, miRmap, miRanda, picTar and TargetScan database. **(B)** The enrichment analysis of KEGG pathways was performed in the 428 genes. **(C)** Three genes (*CDKN1A, MINK1* and *SQSTM1*) were overlapped among the three relevant pathways (Mammary gland, Invasive breast cancer and Cellular responses to stress). **(D-F)** The levels of CDKN1A, MINK1 and SQSTM1 were analyzed in breast cancer (n=1096) and adjacent normal breast tissue (n=111) cohorts from The Cancer Genome Atlas. Statistical significance was determined by the non-parametric Mann-Whitney U test. **(G, H)** The mRNA expressions of CDKN1A, MINK1 and SQSTM1 were detected using qRT-PCR in MDA-MB-231 cells transfected with miR-17-5p mimic, inhibitor or negative control (NC), with GAPDH as a reference. **(I, J)** The mRNA expression of ULBP2 was determined by qRT-PCR in CDKN1A, MINK1 or SQSTM1 knocked-down breast cancer cells, with GAPDH as a reference. The unpaired two-tailed Student’s t-test was used to determine statistical significance in **(G-J)**. *p<0.05, **p<0.01, ***p<0.001 versus NC or si-NC.

Despite evidence suggesting that miR-17-5p can suppress ULBP2 expression, bioinformatics analysis indicates that ULBP2 is not a direct target of miR-17-5p. This implies the existence of a more intricate and indirect regulatory mechanism governing ULBP2 expression by miR-17-5p. To elucidate the connection between miR-17-5p and ULBP2, we initially identified 428 commonly predicted target genes of miR-17-5p across five online databases: PITA, miRmap, miRanda, picTar, and TargetScan (**Figure 3A**). For better understanding the biological features of these genes, we performed KEGG pathway enrichment analysis. The results, presented in **Figure 3B**, revealed that these genes are predominantly associated with several key pathways. Among these pathways, three are particularly relevant to breast cancer and ULBP2 regulation: mammary gland, invasive breast cancer, and cellular responses to stress. As shown in the Venn diagram, three genes (*CDKN1A, MINK1* and *SQSTM1*) were overlapped in the three pathways (**Figure 3C**). Then, differential expression analysis of the three predicted genes were performed between breast cancer (BC) tissues and adjacent normal breast tissues using data from TCGA. The analysis revealed differential expression patterns of the three predicted genes between BC tissues and adjacent normal breast tissues. CDKN1A and MINK1 were both downregulated in BC, with median TPM (Transcripts Per Million) values decreasing from 5925.963 in normal tissue to 4547.018 in BC tissue for CDKN1A, and from 5896.905 in normal tissue to 4666.032 in BC tissue for MINK1. Conversely, SQSTM1 exhibited an opposite expression pattern, with its median TPM value increasing from 11834.51 in normal tissue to 14738.95 in BC tissue (**Figures 3D–F**). This indicates that CDKN1A and MINK1 are downregulated in breast cancer relative to normal tissue, while SQSTM1 shows upregulation in the same comparison. MDA-MB-231 cells were transfected with miR-17-5p mimics or inhibitors, after which the expression levels of the three genes were assessed using qRT-PCR. As shown in **Figures 3G–H**, the expression levels of these genes were found to be inversely correlated with miR-17-5p levels.

To explore the roles the three predicted genes played in ULBP2 regulation, their corresponding siRNAs were constructed and respectively transfected into breast cancer cells. Knockdown of CDKN1A, MINK1 or SQSTM1 in MDA-MB-231 cells led to reduced expression of ULBP2 (**Figure 3I**). For MCF7 cells, ULBP2 expression was downregulated in the MINK1 siRNA-transfected group but upregulated in the CDKN1A and SQSTM1 siRNA-transfected groups. This pattern of ULBP2 expression modulation was not consistent with that observed in MDA-MB-231 cells (**Figure 3J**). The findings demonstrate that the regulation of ULBP2 expression by CDKN1A and SQSTM1 may be cell-specific. In both MDA-MB-231 and MCF7 cells, MINK1 knockdown resulted in the inhibition of ULBP2 expression, leading to its selection for further study. Si-MINK1-2, exhibiting superior efficacy in ULBP2 inhibition compared to si-MINK1-1, was hence chosen for further investigations.

### 3.4 MiR-17-5p downregulates ULBP2 expression by directly binding to 3’-UTR of *MINK1*

**Figure 4:**
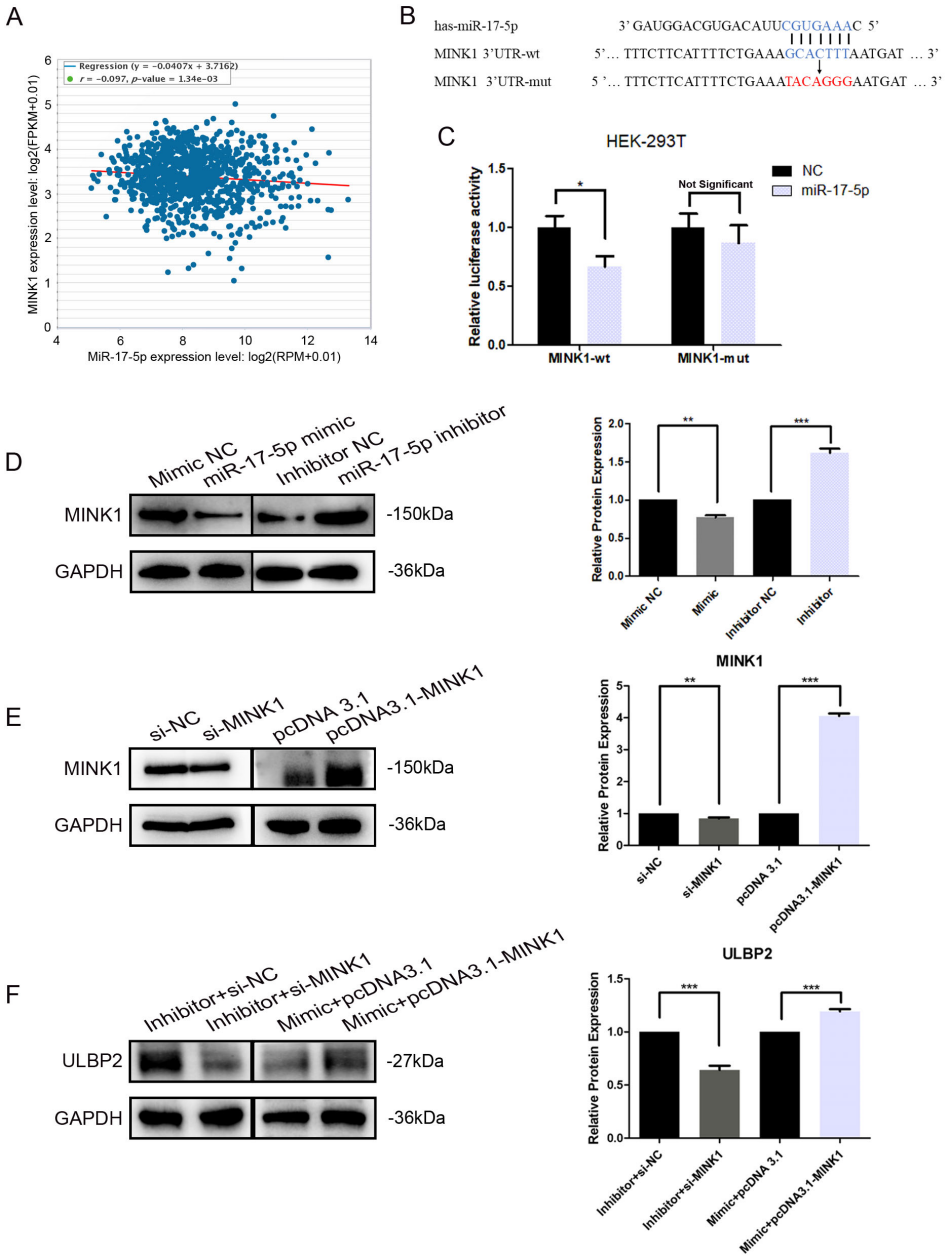
*MINK1* is the target gene of miR-17-5p and promotes ULBP2 expression. **(A)** The correlation between miR-17-5p and MINK1 in 1085 breast tissue samples from TCGA. Linear regression analysis was used to assess the relationship between miR-17-5p and MINK1 expression. **(B)** Schematic representation of predicted miR-17-5p binding sites in the 3’-UTR of *MINK1* and 3’-UTR mutated alignment. **(C)** The dual luciferase assays were performed in HEK-293 T cells. **(D)** MDA-MB-231 cells were transfected with 50 nM of miR-17-5p inhibitor, mimic or negative control (NC) for 24 hours. The MINK1 protein levels were detected by western blot. **(E)** The efficiency of MINK1 knockdown and overexpression was confirmed by western blot. **(F)** The effects of MINKI on ULBP2 expression were assessed using western blot in miR-17-5p exogenously expressed or suppressed MDA-MB-231 cells. **(D-F)** GAPDH was used as a reference in western blot assays and the blots were further quantified with ImageJ (right). The unpaired two-tailed Student’s t-test was used to determine statistical significance. *p<0.05, **p<0.01, ***p<0.001.

Further analyses and studies were conducted to more comprehensively elucidate the regulation of MINK1 by miR-17-5p. An inverse correlation between endogenous miR-17-5p and MINK1 was demonstrated in breast cancer specimens using StarBase analysis (https://starbase.sysu.edu.cn/), suggesting a potential role for miR-17-5p in modulating MINK1 expression in breast cancer patients (**Figure 4A**). To validate the direct binding of miR-17-5p to 3′-UTRs of *MINK1*, a dual-luciferase reporter assay was conducted. The assay showed that miR-17-5p significantly suppressed the luciferase activity of constructs harboring the wild-type *MINK1* 3′-UTR, whereas transfection with miR-17-5p mimic did not produce any appreciable alteration in the luciferase activity of reporters containing mutated *MINK1* 3′-UTR sequences (**Figures 4B, C**). These findings confirm that miR-17-5p suppresses MINK1 expression by directly binding to its 3′-UTR. Overexpression of miR-17-5p caused a marked downregulation of MINK1 protein levels in MDA-MB-231 cells, whereas suppression of miR-17-5p led to a significant upregulation of MINK1 protein levels (**Figure 4D**).

To investigate the effect of MINK1 on ULBP2, we first constructed siRNA (si-MINK1) for MINK1 knockdown and an overexpression vector (pcDNA3.1-MINK1) for MINK1 upregulation. The efficiencies of these constructs were confirmed by Western blot analysis (**Figure 4E**). Then, MDA-MB-231 cells were co-transfected with si-MINK1, pcDNA3.1-MINK1, miR-17-5p mimics, inhibitors or respective controls. As shown in **Figure 4F**, si-MINK1 abrogated the increase in ULBP2 expression induced by miR-17-5p inhibitors, whereas pcDNA3.1-MINK1 plasmids effectively counteracted the suppressive impact of miR-17-5p mimics on ULBP2. Taken together, the results indicate the key role MINK1 plays within the pathway through which miR-17-5p downregulates ULBP2.

### 3.5 JNK/c-Jun is involved in ULBP2 regulation mediated by the miR-17-5p/MINK1 axis

**Figure 5:**
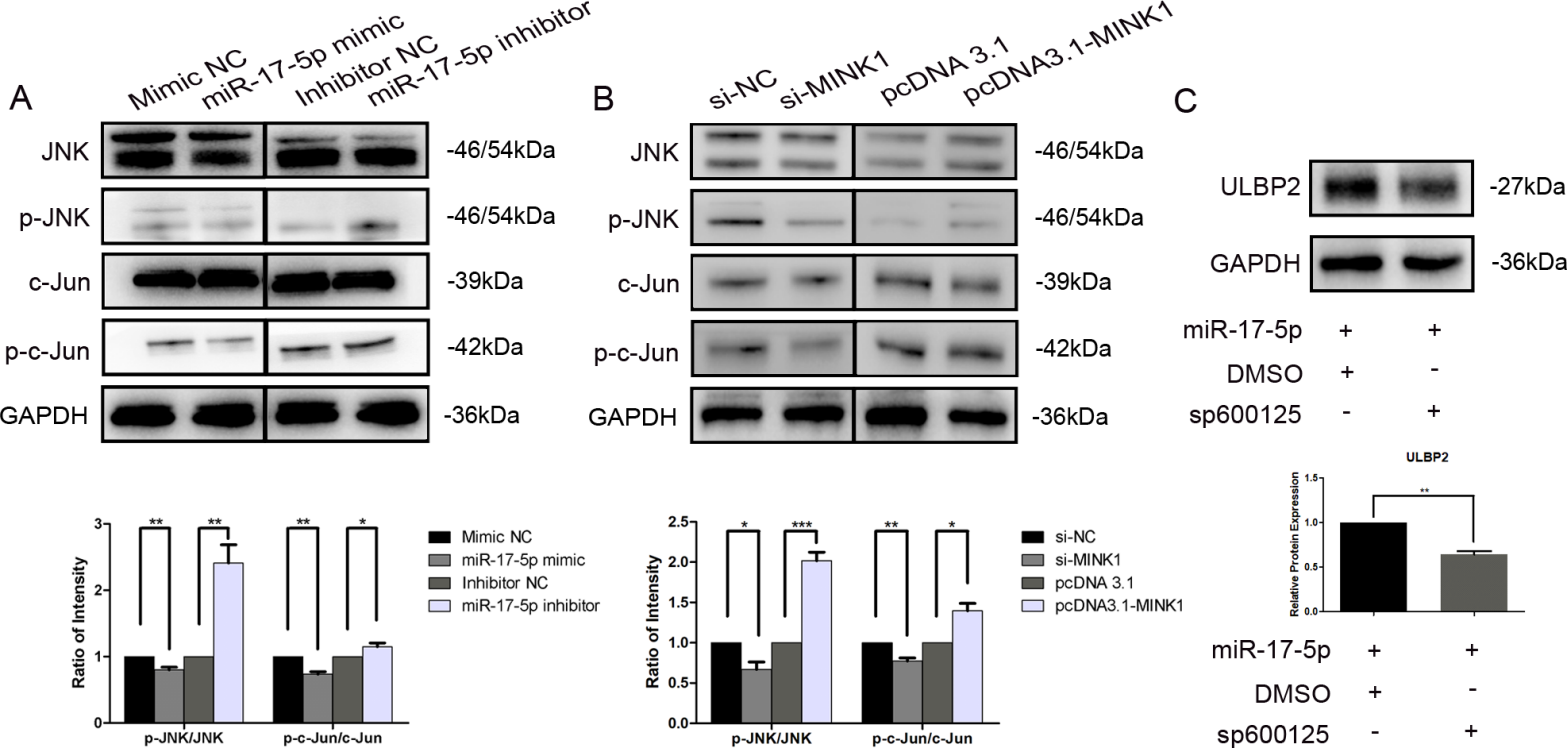
JNK/c-Jun is involved in ULBP2 regulation mediated by the miR-17-5p/MINK1 axis. **(A)** MDA-MB-231 cells were transfected with miR-17-5p inhibitors, mimics or the corresponding controls (NC) for 24 hours. The levels of JNK, p-JNK, c-Jun and p-c-Jun were detected by western blot. **(B)** The expressions of JNK, p-JNK, c-Jun and p-c-Jun were determined in MINK1 knocked-down or overexpressed MDA-MB-231 cells by western blot. **(C)** MDA-MB-231 cells exogenously expressing miR-17-5p were treated with 10 μM of sp600125, a JNK inhibitor, or dimethyl sulfoxide (DMSO, as a vehicle control) for 24 hours. Subsequently, ULBP2 expression was evaluated by Western blot. GAPDH was used as a reference. All the blots were quantified with ImageJ. The unpaired two-tailed Student’s t-test was used to determine statistical significance. *p<0.05, **p<0.01, ***p<0.001.

MINK1, also known as MAP4K6 (Mitogen-activated protein kinase kinase kinase kinase 6), is a serine/threonine kinase that functions as a mitogen-activated protein kinase (MAPK) kinase within the MAPK signaling cascade. Notably, c-Jun N-terminal kinase (JNK) is a prototypical member of the MAPK family. Herein, we explored the involvement of JNK and its downstream target, c-Jun, within the regulation of ULBP2. Western blot analysis demonstrated a significant reduction in the expression levels of phosphorylated JNK (p-JNK, at Thr183/Tyr185) and phosphorylated c-Jun (p-c-Jun, at Ser63) in breast cancer cells that overexpressed miR-17-5p. Conversely, cells transfected with miR-17-5p inhibitors exhibited a notable increase in the levels of these phosphorylated proteins (**Figure 5A**). Knockdown of MINK1 resulted in decreased expression of p-JNK and p-c-Jun, whereas overexpression of MINK1 led to increased levels of these phosphorylated proteins (**Figure 5B**). Furthermore, treatment with sp600125, a specific JNK inhibitor, potentiated the suppressive influence of miR-17-5p on ULBP2 expression (**Figure 5C**). Altogether, the data demonstrate that the JNK/c-Jun pathway contributes to the promotion of ULBP2 expression in breast cancer cells and, to a certain extent, is involved in the mechanism by which miR-17-5p modulates ULBP2.

### 3.6 Resveratrol treatment increases the sensitivity of breast cancer cells to NK cell-mediated lysis both *in vitro* and *in vivo*

**Figure 6:**
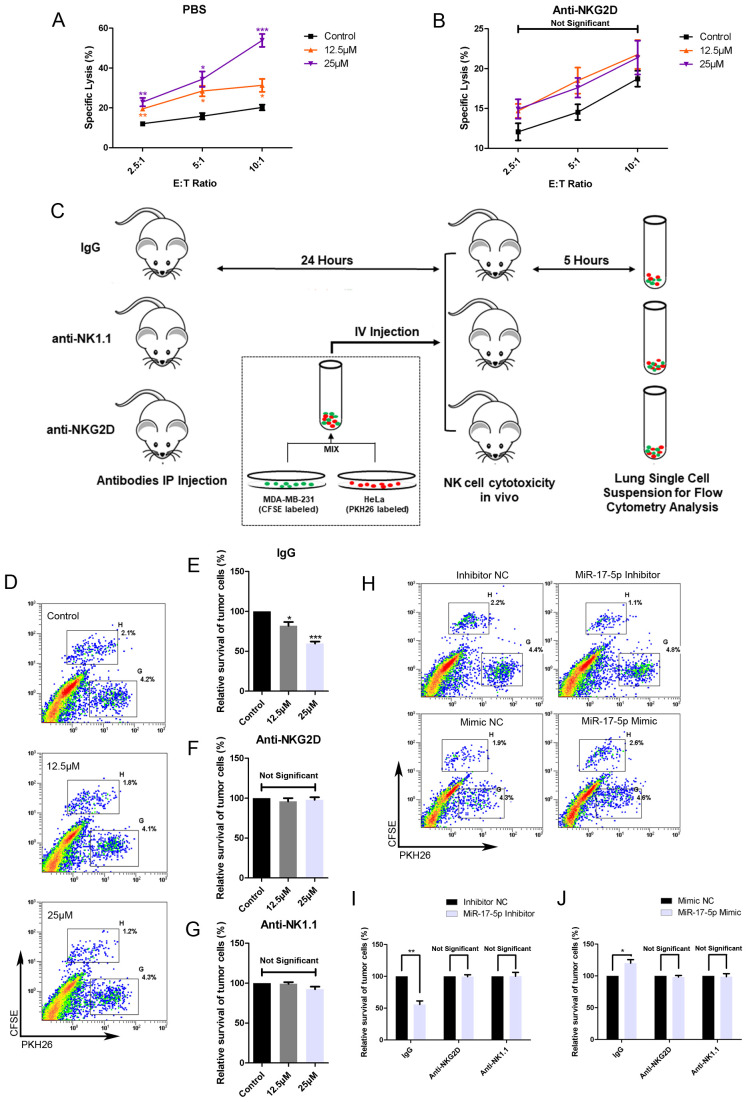
RES contributes to NK mediated cytolysis against breast cancer cells *in vitro* and *in vivo*. **(A, B)** MDA-MB-231 cells were exposed to different concentrations of RES for 48 hours. The NK cell line NK-92MI cells were pretreated with phosphate buffered saline (PBS) **(A)** or anti-NKG2D antibodies **(B)** for 1 hour before the cytotoxicity assay. Cytotoxicity assays were performed with NK-92MI cells as effector cells at different effector-to-target ratios. **(C)** Schematic representation of the *in vivo* experimental procedures. Male C57BL/6 mice were intraperitoneally (IP) injected with 300 μg per mouse of IgG, anti-NKG2D antibody, or anti-NK1.1 antibody. 24 hours later, the mice received an intravenous (IV) injection of a mixture containing [2×10^6^] MDA-MB-231 cells and [2×10^6^] HeLa cells. After 5 hours, the mice were sacrificed, and their lungs were excised and processed to generate single-cell suspensions. **(D-G)** MDA-MB-231 cells were pretreated with different concentrations of RES for 48 hours. Flow cytometry assays were used to analyze the ratios of MDA-MB-231 cells to HeLa cells in lung single-cell suspensions. **(D)** depicts representative results from IgG pretreated groups. **(H-J)** MDA-MB-231 cells were transfected with 50 nM of miR-17-5p mimic, inhibitor or negative control (NC), respectively. Flow cytometry assays were used to analyze the ratios of MDA-MB-231 cells to HeLa cells in lung single-cell suspensions. **(H)** depicts representative results from IgG pretreated groups. Statistical significance was determined by the unpaired two-tailed Student’s t-test. *p<0.05, **p<0.01, ***p<0.001 versus Control.

To assess the effects of RES on the susceptibility of breast cancer cells to clearance by NK cells, MDA-MB-231 cells were exposed to different concentrations of RES for 48 hours. Results from *in vitro* NK cytotoxicity assays at different effector-to-target ratios demonstrated that the lysis of MDA-MB-231 cells by NK cells increased with the concentration of RES pre-treated on the target cells (**Figure 6A**). However, when the NK cells were preconditioned by incubation with an anti-NKG2D blocking antibody, RES exposure induced increase in cancer cell lysis by NK cell would be effectively abolished (**Figure 6B**). This observation points to the critical role of NKG2D receptor recognition and activation in the process.

As illustrated in **Figure 6C**, the *in vivo* cytotoxicity experiments were performed in C57BL/6 mice with HeLa cells as internal control. The survival rate of MDA-MB-231 cells, as determined by the ratio of CFSE-positive cells to PKH26-positive cells, inversely correlated with the concentration of RES used in treatment (**Figures 6D, E**). The anti-NKG2D or anti-NK1.1 antibodies were intraperitoneally injected into mice in the respective groups to block NKG2D receptors or deplete NK cells. In mice pretreated with anti-NKG2D antibodies, the stimulatory effect of RES on the clearance of breast cancer cells by NK cells was abolished (**Figure 6F**). This finding implies that the process is mediated by NKG2D. And similar observations were made in the anti-NK1.1 group, suggesting that NK cells are the primary effectors responsible for the clearance (**Figure 6G**).

Moreover, we investigated the influence of miR-17-5p on tumor cell eradication within an *in vivo* context. MDA-MB-231 cells transfected with miR-17-5p mimics exhibited reduced susceptibility to lysis by immune effector cells. As a result, a lower proportion of cancer cells were eliminated, leading to a higher number of CFSE-labeled cells in the single cell suspension compared to the control group. In contrast, the group transfected with miR-17-5p inhibitors exhibited a lower number of CFSE-labeled cells, indicative of increased elimination of cancer cells. Importantly, all these differences between the groups were completely abolished in mice pretreated with anti-NKG2D and antiNK1.1 antibodies (**Figures 6H–J**).

These results confirm that RES enhances the vulnerability of breast cancer cells to NK cell-mediated cytotoxicity, whereas miR-17-5p functions as a negative regulator of this sensitivity.

### 3.7 Resveratrol increases the level of ULBP2 in xenograft tumors and meanwhile suppresses tumor growth

**Figure 7:**
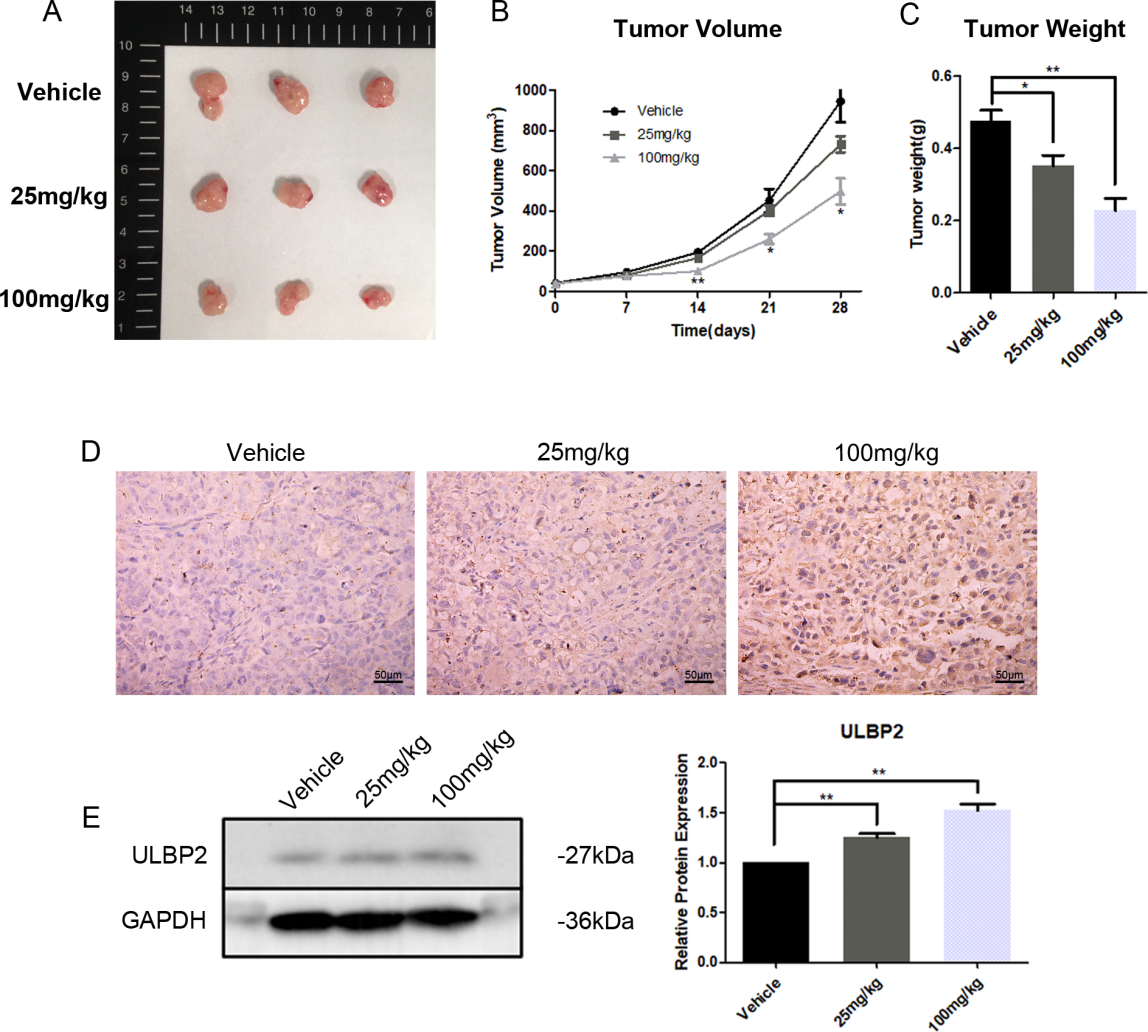
RES suppresses tumor growth and increases ULBP2 expression in xenograft tumors. MDA-MB-231 cells were subcutaneously implanted into female BALB/c (nu/nu) mice. RES or vehicle control was administrated intraperitoneally every day for 28 days. **(A)** The mice were sacrificed for xenograft tumor tissues. **(B)** Tumor volumes were measured every 7 days. **(C)** Tumor weights were measured at the endpoint of the experiment. **(D)** Representative images from immunohistochemistry assay for ULBP2 expression (magnification ×400). **(E)** Levels of ULBP2 protein were examined by western blot with GAPDH as a reference and the blots were quantified with ImageJ. Statistical significance was determined by the unpaired two-tailed Student’s t-test. *p<0.05, **p<0.01 versus Vehicle.

To further investigate the *in vivo* effects of RES on breast cancer growth and ULBP2 expression, female BALB/c (nu/nu) mice were subjected to subcutaneous implantation of MDA-MB-231 cells to establish xenograft tumors. Thereafter, RES was administered intraperitoneally at doses of 0, 25, or 100 mg/kg per day. At the conclusion of a 28-day treatment period, the mice were euthanized, and their tumors were excised and weighed. A marked, dose-dependent reduction in both tumor volume and weight was observed in the RES-treated groups compared to the control group (**Figures 7A–C**). Moreover, IHC and western blot analyses confirmed RES promoted ULBP2 expression in xenograft tumors, consistent with the findings obtained from breast cancer cell lines *in vitro* (**Figures 7D, E**). The data indicate that RES upregulates ULBP2 expression in breast cancer cells and concurrently suppresses tumor growth in an *in vivo* setting.

## 4 Discussion

The study of natural compounds as disease preventive agents or alternatives to synthetic molecules for therapeutic use has become an important subject of interest in recent decades. RES, one of the most representative compounds, has gained the focus of a variety of researches in chemistry and medicine (24). RES exhibits excellent tolerability in both experimental animals and humans. No obvious adverse effects were observed in dogs administered RES orally at a dose of 600 mg/kg/day for 90 days (25). In a subset of healthy volunteers administered oral RES at a high dose of 2000 mg twice daily, only mild-to-moderate gastrointestinal disturbances were reported (26). RES performs multiple health-promoting activities, including anticancer. Breast cancer, the most prevalent malignancy among females globally, threatens human health seriously and imposes a heavy burden on patients, their families and society (27, 28). A growing body of research increasingly substantiates RES’s potential in breast cancer prophylaxis and therapy. This includes mechanisms such as the inhibition of angiogenesis, suppression of cell migration and metastasis, induction of cell cycle arrest and apoptosis, as well as modulation of epigenetic processes (7, 29–31). Consistently, the present study revealed that administering RES to mice at doses of either 25 mg/kg/day or 100 mg/kg/day for a 28-day period was well-tolerated and effectively inhibited cancer growth.

Considering the critical role that ULBP2 plays in facilitating cancer cell elimination by NK cells and the immune-modulatory effects exerted by RES, the current research aimed to elucidate the impact of RES on ULBP2 expression and the underlying molecular mechanisms involved. RES was demonstrated to augment ULBP2 expression in four breast cancer cell lines. It heightened the susceptibility of MDA-MB-231 cells to NK cell-mediated cytotoxicity *in vitro*. However, the increased susceptibility was diminished when cytolysis was performed with NK cells whose NKG2D receptors were blocked, suggesting that NKG2D plays a crucial role in this process. Furthermore, *in vivo* experiments demonstrated that RES dose-dependently enhanced the killing of intravenously injected MDA-MB-231 cells in C57BL/6 mice. This effect was eliminated in mice pre-treated with anti-NKG2D and anti-NK1.1 antibodies, suggesting that the *in vivo* cytotoxicity against MDA-MB-231 cells was primarily executed by NKG2D-activated NK cells. These results point to the potential that RES may serve as an enhancer of ULBP2-mediated cancer cell clearance by NK cells. Subsequently, the mechanism by which RES upregulates the expression of ULBP2 was intensively investigated.

MiR-17-5p is upregulated in various types of cancer and is known for its oncogenic properties (32, 33). It drives cancer initiation, progression, and metastasis by promoting cancer cell motility, proliferation, invasiveness, angiogenesis and chemoresistance (34–37). Accordantly, findings from our previous research indicated that miR-17-5p plays a pivotal role in promoting the epithelial-mesenchymal transition, thereby enhancing the migratory and invasive capabilities of breast cancer cells (22). In the current study, the exposure to RES led to a dose-dependent reduction in miR-17-5p levels in breast cancer cells. MiR-17-5p has been documented to suppress the expression of MICA and MICB, two other important NKG2DLs, in hepatocellular and colorectal cancers (38, 39). However, the potential regulatory role of miR-17-5p in modulating ULBP2 expression has not yet been reported. Herein, miR-17-5p was demonstrated to suppress ULBP2 expression in breast cancer line MDA-MB-231 and MCF7 cells. And its effect on the susceptibility of MDA-MB-231 cells to NK cell-mediated cytolysis was evaluated in mouse model. MDA-MB-231 cells transfected with miR-17-5p mimics exhibited reduced susceptibility to lysis by murine NK cells, while the cells transfected with miR-17-5p inhibitors showed higher sensitivity to NK cell killing *in vivo*. However, these changes in susceptibility induced by miR-17-5p upregulation or downregulation were abolished in mice pretreated with anti-NKG2D and anti-NK1.1 antibodies. Anti-NKG2D antibodies block NKG2D receptors, while anti-NK1.1 antibodies deplete NK cells. These findings suggest that both NK cells and their activating receptor NKG2D play a critical role in mediating cytotoxicity against breast cancer cells in mice. Additionally, the regulatory role of miR-17-5p in modulating ULBP2 expression, a ligand for NKG2D, was confirmed. This further indicates that miR-17-5p influences the recognition and killing of breast cancer cells by NK cells through its effect on ULBP2 expression.

But bioinformatic analysis did not identify any predicted miR-17-5p binding sites within the 3′-UTR of *ULBP2*, indicating that *ULBP2* was not a direct target gene of miR-17-5p. Consequently, we focused on screening potential target genes of miR-17-5p in the context of ULBP2 regulation. Our efforts led to the prediction that *MINK1* is the most probable target gene. An inverse correlation between MINK1 expression and miR-17-5p levels was observed across 1085 breast cancer specimens in the TCGA dataset. Cellular MINK1 levels were diminished upon transfection with miR-17-5p mimics and augmented following treatment with miR-17-5p inhibitors, respectively. using a dual-luciferase reporter assay, we demonstrated that miR-17-5p inhibited MINK1 expression by directly interacting with its 3′-UTR, thereby validating their direct targeting relationship. The exogenous expression of MINK1 effectively counteracted the decrease in ULBP2 protein levels induced by miR-17-5p overexpression, thus demonstrating the positive regulatory role of MINK1 on ULBP2 expression.

MINK1, an integral member of the mammalian Ste20-like serine/threonine kinase family, functions as a MAPK kinase in regulating diverse cellular processes such as senescence, motility, and chemoresistance (40–42). The MAPK pathway is a well characterized signaling pathway which controls a multitude of fundamental cellular processes, including inflammation, differentiation, apoptosis, proliferation, and others (43). But its role in NKG2DL regulation has not yet been well defined. Extracellular signal regulated kinase (ERK), a component of MAPK signaling cascade, was verified to promote ULBP2 expression in breast cancer in our previous research, which indicated the involvement of MAPK signaling in NKG2DL regulation (15). The JNK/c-Jun pathway is one of the classical MAPK pathways (44). In this work, the activation of JNK and its downstream effector molecule c-Jun was attenuated in breast cancer cells overexpressing miR-17-5p or subjected to MINK1 knockdown, whereas miR-17-5p downregulation or MINK1 upregulation led to opposing outcomes. Moreover, the JNK inhibitor sp600125 potentiated the suppressive effect of miR-17-5p on ULBP2 expression. These findings confirmed the involvement of JNK/c-Jun MAPK signaling cascade in ULBP2 regulation. It is reasonable to ask how the JNK/c-Jun pathway contributes to ULBP2 expression. C-Jun, a well-characterized transcription factor primarily activated by the JNK pathway, plays a crucial role in multiple biological processes by binding to specific DNA sequences to initiate transcriptional activity (45, 46). Due to this, ULBP2 expression might be regulated by c-Jun directly or indirectly. However, this possibility requires further exploration. Moreover, RES exhibited suppression of xenograft tumor growth in mice. Although the underlying mechanisms warrant further exploration, it is speculated that the downregulation of miR-17-5p, given its oncogenic properties, may contribute, at least in part, to the inhibitory effect of RES on breast cancer growth in murine models.

Considering the established capacity of RES to enhance NK cell-mediated cytotoxicity against breast cancer cells, further preclinical investigations are warranted to evaluate the administration route, optimal dosage, and treatment duration to maximize therapeutic efficacy while minimizing potential toxicity. Whether RES impacts ULBP2 expression in normal cells warrants further investigation. Additionally, methods to improve the bioavailability of RES should be developed. Moreover, integrating RES with conventional breast cancer treatment regimens deserves further exploration to leverage potential synergistic benefits.

## 5 Conclusion

**Figure 8:**
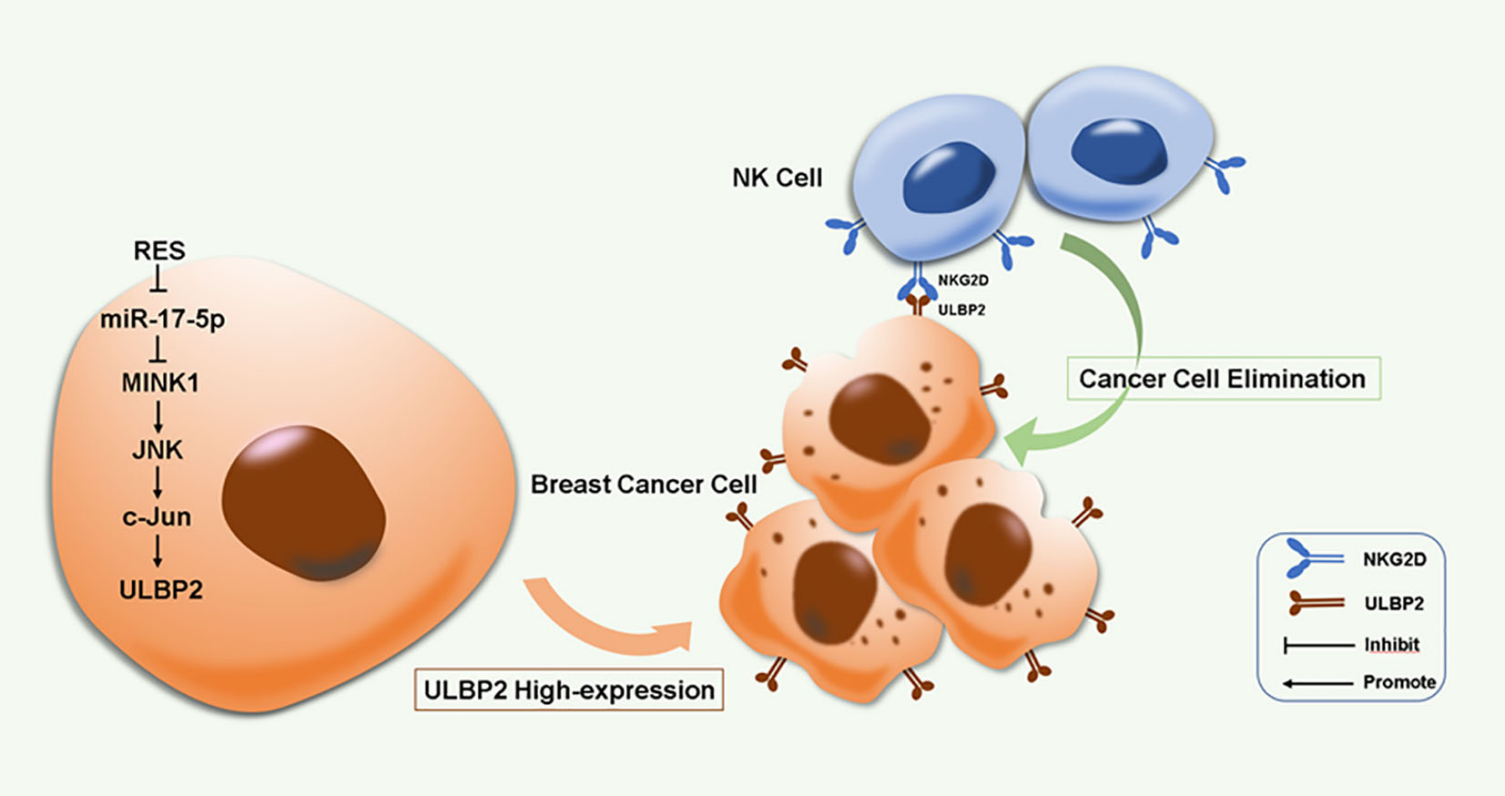
A proposed model for RES induced immune elimination of breast cancer cells by NK cells via miR-17-5p/MINK1/JNK/c-Jun/ULBP2 cascade.

RES induces ULBP2 expression and consequently enhances breast cancer cell elimination by NK cells through the suppression of miR-17-5p and activation of the MINK1/JNK/c-Jun cascade. This represents a novel biological mechanism in ULBP2 regulation (**Figure 8**). Our work suggests that RES has the potential to be an effective therapeutic agent for inhibiting breast cancer progression and enhancing NK cell-based cancer immunotherapy.

## Data Availability

The raw data supporting the conclusions of this article will be made available by the authors, without undue reservation.

## References

[1] Brockmueller A Sajeev A Koklesova L Samuel SM Kubatka P Büsselberg D. Resveratrol as sensitizer in colorectal cancer plasticity. Cancer metastasis Rev. (2023) 43:55–85. doi: 10.1007/s10555-023-10126-x PMC11016130 37507626

[2] Breuss JM Atanasov AG Uhrin P. Resveratrol and its effects on the vascular system. Int J Mol Sci. (2019) 20:1523. doi: 10.3390/ijms20071523 PMC6479680 30934670

[3] Brockmueller A Buhrmann C Shayan P Shakibaei M. Resveratrol induces apoptosis by modulating the reciprocal crosstalk between p53 and Sirt-1 in the CRC tumor microenvironment. Front Immunol. (2023) 14:1225530. doi: 10.3389/fimmu.2023.1225530 37575245 PMC10413256

[4] Hankittichai P Thaklaewphan P Wikan N Ruttanapattanakul J Potikanond S Smith DR. Resveratrol Enhances Cytotoxic Effects of Cisplatin by Inducing Cell Cycle Arrest and Apoptosis in Ovarian Adenocarcinoma SKOV-3 Cells through Activating the p38 MAPK and Suppressing AKT. Pharm (Basel Switzerland). (2023) 16:755. doi: 10.3390/ph16050755 PMC10220637 37242538

[5] Song B Wang W Tang X Goh RMW Thuya WL Ho PCL. Inhibitory potential of resveratrol in cancer metastasis: from biology to therapy. Cancers. (2023) 15:2758. doi: 10.3390/cancers15102758 37345095 PMC10216034

[6] Selvakumar P Badgeley A Murphy P Anwar H Sharma U Lawrence K. Flavonoids and other polyphenols act as epigenetic modifiers in breast cancer. Nutrients. (2020) 12:761. doi: 10.3390/nu12030761 32183060 PMC7146477

[7] Sun Y Zhou QM Lu YY Zhang H Chen QL Zhao M. Resveratrol inhibits the migration and metastasis of MDA-MB-231 human breast cancer by reversing TGF-beta1-induced epithelial-mesenchymal transition. Molecules. (2019) 24:1131. doi: 10.3390/molecules24061131 30901941 PMC6471699

[8] Kowsari H Davoodvandi A Dashti F Mirazimi SMA Bahabadi ZR Aschner M. Resveratrol in cancer treatment with a focus on breast cancer. Curr Mol Pharmacol. (2023) 16:346–61. doi: 10.2174/1874467215666220616145216 35713130

[9] Chen L Musa AE. Boosting immune system against cancer by resveratrol. Phytotherapy research: PTR. (2021) 35:5514–26. doi: 10.1002/ptr.7189 34101276

[10] Delmas D Hermetet F Aires V. PD-1/PD-L1 checkpoints and resveratrol: A controversial new way for a therapeutic strategy. Cancers. (2021) 13:4509. doi: 10.3390/cancers13184509 34572736 PMC8467857

[11] Schmiedel D Mandelboim O. NKG2D ligands-critical targets for cancer immune escape and therapy. Front Immunol. (2018) 9:2040. doi: 10.3389/fimmu.2018.02040 30254634 PMC6141707

[12] Campos-Silva C Kramer MK Valés-Gómez M. NKG2D-ligands: Putting everything under the same umbrella can be misleading. Hla. (2018) 91:489–500. doi: 10.1111/tan.13246 29521021

[13] Yang C Qian C Zheng W Dong G Zhang S Wang F. Ginsenoside Rh2 enhances immune surveillance of natural killer (NK) cells via inhibition of ERp5 in breast cancer. Phytomedicine: Int J phytotherapy phytopharmacology. (2024) 123:155180. doi: 10.1016/j.phymed.2023.155180 38043385

[14] Jin F Wu Z Hu X Zhang J Gao Z Han X. The PI3K/Akt/GSK-3β/ROS/eIF2B pathway promotes breast cancer growth and metastasis via suppression of NK cell cytotoxicity and tumor cell susceptibility. Cancer Biol Med. (2019) 16:38–54. doi: 10.20892/j.issn.2095-3941.2018.0253 31119045 PMC6528454

[15] Shen J Pan J Du C Si W Yao M Xu L. Silencing NKG2D ligand-targeting miRNAs enhances natural killer cell-mediated cytotoxicity in breast cancer. Cell Death Dis. (2017) 8:e2740. doi: 10.1038/cddis.2017.158 28383557 PMC5477582

[16] Wu HY Li KX Pan WY Guo MQ Qiu DZ He YJ. Venetoclax enhances NK cell killing sensitivity of AML cells through the NKG2D/NKG2DL activation pathway. Int immunopharmacology. (2022) 104:108497. doi: 10.1016/j.intimp.2021.108497 34999394

[17] Zhao P Sun X Li H Liu Y Cui Y Tian L. c-myc targets HDAC3 to suppress NKG2DL expression and innate immune response in N-type SCLC through histone deacetylation. Cancers. (2022) 14:457. doi: 10.3390/cancers14030457 35158730 PMC8833590

[18] Alkhayer R Ponath V Frech M Adhikary T Graumann J Neubauer A. KLF4-mediated upregulation of the NKG2D ligand MICA in acute myeloid leukemia: a novel therapeutic target identified by enChIP. Cell communication signaling: CCS. (2023) 21:94. doi: 10.1186/s12964-023-01118-z 37143070 PMC10157933

[19] Ullrich E Koch J Cerwenka A Steinle A. New prospects on the NKG2D/NKG2DL system for oncology. Oncoimmunology. (2013) 2:e26097. doi: 10.4161/onci.26097 24353908 PMC3862635

[20] Allende-Vega N Marco Brualla J Falvo P Alexia C Constantinides M de Maudave AF. Metformin sensitizes leukemic cells to cytotoxic lymphocytes by increasing expression of intercellular adhesion molecule-1 (ICAM-1). Sci Rep. (2022) 12:1341. doi: 10.1038/s41598-022-05470-x 35079096 PMC8789909

[21] Vasu S He S Cheney C Gopalakrishnan B Mani R Lozanski G. Decitabine enhances anti-CD33 monoclonal antibody BI 836858-mediated natural killer ADCC against AML blasts. Blood. (2016) 127:2879–89. doi: 10.1182/blood-2015-11-680546 PMC4900955 27013443

[22] Bao C Liu T Qian L Xiao C Zhou X Ai H. Shikonin inhibits migration and invasion of triple-negative breast cancer cells by suppressing epithelial-mesenchymal transition via miR-17-5p/PTEN/Akt pathway. J Cancer. (2021) 12:76–88. doi: 10.7150/jca.47553 33391404 PMC7738816

[23] Boissel N Rea D Tieng V Dulphy N Brun M Cayuela JM. BCR/ABL oncogene directly controls MHC class I chain-related molecule A expression in chronic myelogenous leukemia. J Immunol (Baltimore Md: 1950). (2006) 176:5108–16. doi: 10.4049/jimmunol.176.8.5108 16585609

[24] Duta-Bratu CG Nitulescu GM Mihai DP Olaru OT. Resveratrol and other natural oligomeric stilbenoid compounds and their therapeutic applications. Plants (Basel Switzerland). (2023) 12:2935. doi: 10.3390/plants12162935 37631147 PMC10459741

[25] Johnson WD Morrissey RL Usborne AL Kapetanovic I Crowell JA Muzzio M. Subchronic oral toxicity and cardiovascular safety pharmacology studies of resveratrol, a naturally occurring polyphenol with cancer preventive activity. Food Chem Toxicol. (2011) 49:3319–27. doi: 10.1016/j.fct.2011.08.023 PMC3223276 21939727

[26] la Porte C Voduc N Zhang G Seguin I Tardiff D Singhal N. Steady-State pharmacokinetics and tolerability of trans-resveratrol 2000 mg twice daily with food, quercetin and alcohol (ethanol) in healthy human subjects. Clin Pharmacokinet. (2010) 49:449–54. doi: 10.2165/11531820-000000000-00000 20528005

[27] Siegel RL Miller KD Wagle NS Jemal A. Cancer statistics, 2023. CA: Cancer J Clin. (2023) 73:17–48. doi: 10.3322/caac.21763 36633525

[28] Xia C Dong X Li H Cao M Sun D He S. Cancer statistics in China and United States, 2022: profiles, trends, and determinants. Chin Med J. (2022) 135:584–90. doi: 10.1097/cm9.0000000000002108 PMC8920425 35143424

[29] Liang ZJ Wan Y Zhu DD Wang MX Jiang HM Huang DL. Resveratrol mediates the apoptosis of triple negative breast cancer cells by reducing POLD1 expression. Front Oncol. (2021) 11:569295. doi: 10.3389/fonc.2021.569295 33747905 PMC7970754

[30] Izquierdo-Torres E Hernandez-Oliveras A Meneses-Morales I Rodriguez G Fuentes-Garcia G Zarain-Herzberg A. Resveratrol up-regulates ATP2A3 gene expression in breast cancer cell lines through epigenetic mechanisms. Int J Biochem Cell Biol. (2019) 113:37–47. doi: 10.1016/j.biocel.2019.05.020 31173924

[31] Farghadani R Naidu R. The anticancer mechanism of action of selected polyphenols in triple-negative breast cancer (TNBC). Biomedicine pharmacotherapy = Biomedecine pharmacotherapie. (2023) 165:115170. doi: 10.1016/j.biopha.2023.115170 37481930

[32] Pidíková P Herichová I. miRNA clusters with up-regulated expression in colorectal cancer. Cancers. (2021) 13:2979. doi: 10.3390/cancers13122979 34198662 PMC8232258

[33] Moi L Braaten T Al-Shibli K Lund E Busund LR. Differential expression of the miR-17-92 cluster and miR-17 family in breast cancer according to tumor type; results from the Norwegian Women and Cancer (NOWAC) study. J Trans Med. (2019) 17:334. doi: 10.1186/s12967-019-2086-x PMC6775665 31581940

[34] Sun K Chen L Li Y Huang B Yan Q Wu C. METTL14-dependent maturation of pri-miR-17 regulates mitochondrial homeostasis and induces chemoresistance in colorectal cancer. Cell Death Dis. (2023) 14:148. doi: 10.1038/s41419-023-05670-x 36810285 PMC9944299

[35] Zhang Y Wang S Lai Q Fang Y Wu C Liu Y. Cancer-associated fibroblasts-derived exosomal miR-17-5p promotes colorectal cancer aggressive phenotype by initiating a RUNX3/MYC/TGF-β1 positive feedback loop. Cancer Lett. (2020) 491:22–35. doi: 10.1016/j.canlet.2020.07.023 32730779

[36] Kim TW Lee YS Yun NH Shin CH Hong HK Kim HH. MicroRNA-17-5p regulates EMT by targeting vimentin in colorectal cancer. Br J cancer. (2020) 123:1123–30. doi: 10.1038/s41416-020-0940-5 PMC7524803 32546833

[37] Song J Liu Y Wang T Li B Zhang S. MiR-17-5p promotes cellular proliferation and invasiveness by targeting RUNX3 in gastric cancer. Biomedicine pharmacotherapy = Biomedecine pharmacotherapie. (2020) 128:110246. doi: 10.1016/j.biopha.2020.110246 32447210

[38] Wu J Zhang XJ Shi KQ Chen YP Ren YF Song YJ. Hepatitis B surface antigen inhibits MICA and MICB expression via induction of cellular miRNAs in hepatocellular carcinoma cells. Carcinogenesis. (2014) 35:155–63. doi: 10.1093/carcin/bgt268 23917076

[39] Qian M Geng J Luo K Huang Z Zhang Q Zhang JA. BCL11B regulates MICA/B-mediated immune response by acting as a competitive endogenous RNA. Oncogene. (2020) 39:1514–26. doi: 10.1038/s41388-019-1083-0 31673069

[40] Mohanty S Mohapatra P Shriwas O Ansari SA Priyadarshini M Priyadarsini S. CRISPR-based kinome-screening revealed MINK1 as a druggable player to rewire 5FU-resistance in OSCC through AKT/MDM2/p53 axis. Oncogene. (2022) 41:4929–40. doi: 10.1038/s41388-022-02475-8 PMC9630125 36182968

[41] Nicke B Bastien J Khanna SJ Warne PH Cowling V Cook SJ. Involvement of MINK, a Ste20 family kinase, in Ras oncogene-induced growth arrest in human ovarian surface epithelial cells. Mol Cell. (2005) 20:673–85. doi: 10.1016/j.molcel.2005.10.038 16337592

[42] Chuang HC Wang X Tan TH. MAP4K family kinases in immunity and inflammation. Adv Immunol. (2016) 129:277–314. doi: 10.1016/bs.ai.2015.09.006 26791862

[43] Burgermeister E. Mitogen-activated protein kinase and exploratory nuclear receptor crosstalk in cancer immunotherapy. Int J Mol Sci. (2023) 24:14546. doi: 10.3390/ijms241914546 37833991 PMC10572424

[44] Hammouda MB Ford AE Liu Y Zhang JY. The JNK signaling pathway in inflammatory skin disorders and cancer. Cells. (2020) 9:857. doi: 10.3390/cells9040857 32252279 PMC7226813

[45] Fan F Podar K. The role of AP-1 transcription factors in plasma cell biology and multiple myeloma pathophysiology. Cancers. (2021) 13:2326. doi: 10.3390/cancers13102326 34066181 PMC8151277

[46] Hasanpourghadi M Pandurangan AK Mustafa MR. Modulation of oncogenic transcription factors by bioactive natural products in breast cancer. Pharmacol Res. (2018) 128:376–88. doi: 10.1016/j.phrs.2017.09.009 28923544

