# Supplementary material for: Resveratrol contributes to NK cell-mediated breast cancer cytotoxicity by upregulating ULBP2 through miR-17-5p downmodulation and activation of MINK1/JNK/c-Jun signaling
Source: Front Immunol. 2025 Feb 3;16:1515605. doi: 10.3389/fimmu.2025.1515605 (PMC11830804; doi:10.3389/fimmu.2025.1515605)
Supplement: Supplementary file 1 [file DataSheet1.pdf]

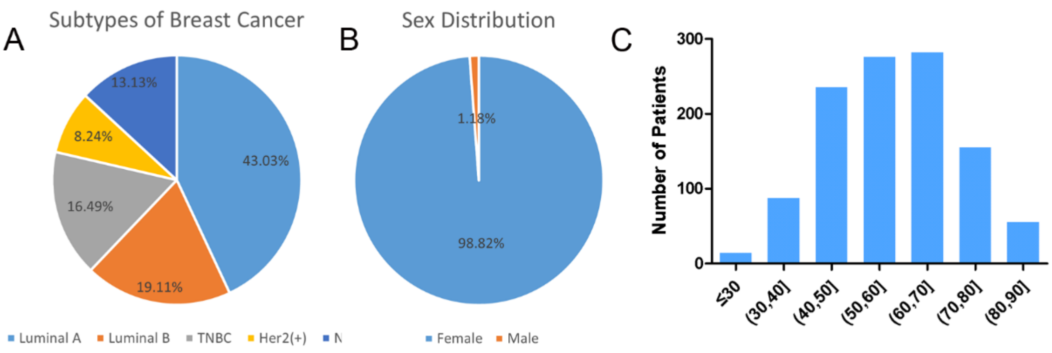

Supplementary Figure 1. Patient Characteristics of Breast Cancer Samples Archived from TCGA. (A) Subtypes of breast cancer. (B) Sex distribution of the breast cancer patients. (C) Age at diagnosis of the patients. The age brackets are defined as follows: (30,40] indicates ages greater than 30 and up to 40.
